# Vernodalin and *Gymnanthemum extensum* Crude Extracts Exhibit In Vitro Anticancer Activity with Differential Regulation of Cancer-Associated Signaling Proteins in Breast and Ovarian Cancer Cells

**DOI:** 10.3390/biomedicines14061331

**Published:** 2026-06-11

**Authors:** Muhammad Faisal, Yaowapa Sukpondma, Juntakarn Sangket, Siriporn Taraporn, Sirinapa Dokduang, Potchanapond Graidist

**Affiliations:** 1 Department of Biomedical Sciences and Biomedical Engineering, Faculty of Medicine, Prince of Songkla University, Hat Yai 90110, Thailand; muhammadfaisal@umsu.ac.id (M.F.); sjuntaka@medicine.psu.ac.th (J.S.); tarsirip@medicine.psu.ac.th (S.T.); dsirinap@medicine.psu.ac.th (S.D.); 2 Department of Pharmacology, Faculty of Medicine, Universitas Muhammadiyah Sumatera Utara, Medan 20217, Indonesia; 3 Division of Physical Science and Center of Excellence for Innovation in Chemistry, Faculty of Science, Prince of Songkla University, Hat Yai 90110, Thailand; yaowapa.suk@psu.ac.th

**Keywords:** anticancer, breast cancer, *Gymnanthemum extensum*, ovarian cancer, vernodalin

## Abstract

**Background/Objectives**: Vernodalin (VD) and crude extracts from *Gymnanthemum extensum* leaves have previously demonstrated anticancer activity; however, their underlying molecular effects remain incompletely understood. This study investigated the anticancer activities of VD and *G. extensum* extracts and characterized their associated molecular responses in breast (MDA-MB-231) and ovarian (A2780) cancer cells. **Methods**: *G. extensum* leaves were extracted with dichloromethane and ethyl acetate to obtain DEGE and EAGE, respectively. VD was isolated from EAGE and characterized by ^1^H-NMR and HPLC. Phytochemical profiles of the extracts were analyzed by GC-MS and HPLC. Cytotoxicity, clonogenic survival, cell cycle progression, migration, and protein expression were evaluated using MTT assay, colony formation assay, flow cytometry, wound healing assay, and Western blotting. **Results**: GC–MS analysis revealed distinct phytochemical compositions between DEGE and EAGE, although both extracts contained high levels of neophytadiene and phytol. VD, DEGE, and EAGE inhibited cell proliferation and migration in both cancer cell lines. VD suppressed proteins associated with cancer progression, including SMYD3, BRAF, MELK, FOXM1, Cyclin B1, MDR1/ABCB1, and MMP-9, with molecular responses differing between MDA-MB-231 and A2780 cells. DEGE and EAGE exhibited molecular regulatory patterns distinct from those of purified VD, suggesting contributions from multiple phytochemical constituents. **Conclusions**: VD and *G. extensum* crude extracts exhibit significant in vitro anticancer activity against breast and ovarian cancer cells and induce distinct molecular responses. The differential effects of DEGE and EAGE may be attributable to differences in their phytochemical constituents.

## 1. Introduction

In 2022, breast cancer was reported as the most prevalent malignancy among women, with nearly 2.5 million new cases and approximately 650,000 deaths. Moreover, ovarian cancer is less common; it has a disproportionately high mortality rate, accounting for over 60% of cases. The incidence of both cancers is expected to rise steadily through 2050 [1,2]. Conventional therapies, particularly chemotherapy, often impose substantial burdens on patients, including severe adverse effects, cumulative toxicity, and the development of drug resistance. These challenges underscore the need for safer and more selective therapeutic strategies, particularly those targeting molecular pathways shared by both cancer types, with the potential to improve survival outcomes while minimizing damage to healthy tissues. In this context, medicinal plants have emerged as promising sources of alternative therapies, offering advantages such as improved safety profiles, affordability, and accessibility compared with conventional chemotherapeutics [3].

*G. extensum*, commonly known as the Bitter Leaf Tree, has a history of use in traditional medicine for managing metabolic disorders, including diabetes, hypertension, hypercholesterolemia, and for supporting liver detoxification. Our previous work demonstrated that dichloromethane and ethyl acetate extracts of *G. extensum* exhibited notable cytotoxic activity against breast cancer cells (Michigan Cancer Foundation-7; MCF-7) and ovarian cancer cells (A2780) [4]. Among the bioactive constituents, vernodalin (VD) has emerged as one of the most potent anticancer compounds in the dichloromethane extract of *G. extensum* leaves [5]. Subsequent studies have further shown that VD exerts strong cytotoxic effects across multiple cancer types, including breast and ovarian cancers, primarily through modulation of key regulators involved in cell survival, cell cycle progression, adhesion, and migration [6,7,8]. Our previous in silico analysis identified heat shock protein 90 alpha (HSP90α), JAK3, IMPDH2, COX-2, AKR1B1, Cyclin B1, MetAP2, and CDC25A as potential molecular targets of VD [9]. Among these proteins, HSP90α emerged as a particularly compelling target because of its central role in maintaining the stability and activity of numerous oncogenic client proteins. Dysregulation of HSP90α has been implicated in cancer cell survival, proliferation, migration, and metastasis, making it an attractive therapeutic target for anticancer drug development [10,11]. Among its isoforms, HSP90α (HSP90AA1) is frequently overexpressed in breast and ovarian cancer cell lines and has been implicated in tumor progression [12,13,14]. Dysregulation of HSP90α can lead to misfolding and proteasomal degradation of its client proteins, resulting in disruption of oncogenic signaling pathways [15].

DEGE and EAGE have previously demonstrated in vitro anticancer activity. In addition, VD, a bioactive constituent of *G. extensum*, has shown promising anticancer potential, while our previous in silico analysis suggested HSP90α and several cancer-related proteins as potential molecular targets [9]. Given the central role of HSP90α in regulating multiple oncogenic signaling pathways, this study investigated the anticancer effects of VD, DEGE, and EAGE in non-aggressive ovarian cancer cells (A2780) and highly aggressive breast cancer cells (MDA-MB-231), with particular focus on alterations in HSP90α-associated protein networks. Cytotoxicity, clonogenic survival, cell cycle progression, migration, and the expression of selected HSP90α-related signaling proteins were evaluated to provide insight into the molecular responses induced by these treatments.

## 2. Materials and Methods

### 2.1. Plant Materials Collection

Leaves of *G. extensum* were collected by Muhammad Faisal in Hat Yai City, Songkhla Province, Thailand (7.01° N, 100.47° E), on 6 September 2022. The plant was taxonomically identified by Professor Suchada Sukrong from the Department of Pharmacognosy and Pharmaceutical Botany, Faculty of Pharmaceutical Sciences, Chulalongkorn University, and deposited in the Museum of Natural Medicine, Faculty of Pharmaceutical Sciences, Chulalongkorn University with a voucher specimen (No. SS-PG-001).

### 2.2. Chemicals and Reagents

In plant extraction, dichloromethane (CH_2_Cl_2_) and ethyl acetate (C_4_H_8_O_2_) were purchased from J.T. Baker® (Phillipsburg, NJ, USA). To maintain the cell lines, Roswell Park Memorial Institute Medium (RPMI-1640) and Dulbecco’s Modified Eagle Medium (DMEM), L-Glutamine, Fetal Bovine Serum (FBS), and Antibiotics (Penicillin-Streptomycin) were purchased from Gibco^TM^ (Waltham, MA, USA). Doxorubicin (DOX), cisplatin (CIS), and tanespimycin (17-AAG) were purchased from Sigma-Aldrich (St. Louis, MO, USA) and assigned as control treatments and an analytical standard. MTT assay, dimethyl sulfoxide (DMSO), and crystal violet were obtained from GibThai (Bangkok, Thailand), Amresco® (Solon, OH, USA), and Merck (Darmstadt, Germany), respectively. Propidium Iodide (PI) was purchased from Cytek Biosciences (Fremont, CA, USA). In the Western Blot study, several primary antibodies against HSP90α, MDR1/ABCB1, and GAPDH, as well as an HRP-conjugated secondary antibody, were purchased from Cell Signaling Technology (Danvers, MA, USA) with catalog no. 8165, 12683, 5174, and 7074/7076, respectively. SMYD3 and MELK primary antibodies were obtained from Abcam (Cambridge, UK) with catalog no. EPR11107(2) and EPR3981, respectively. Cyclin B1 and BRAF primary antibodies were purchased from Santa Cruz Biotechnology (Dallas, TX, USA) with catalog no. sc-7393 and sc-5284, respectively. Meanwhile, MMP-9 primary antibody was obtained from Torrey Pines Biolabs (Secaucus, NJ, USA) with a catalog no. TP221AF. All organic solvents used in the study were specifically used for extraction and pure compound isolation, such as pro-analyst-grade and high-performance liquid chromatography (HPLC)-grade dichloromethane and ethyl acetate.

### 2.3. Extraction and Isolation

#### 2.3.1. Crude Extracts of *G. extensum*

To extract the phytochemical compounds of *G. extensum*, the maceration technique was carried out by adopting our previous study [4]. *G. extensum* leaves in dried powder form (212.8 g) were successively extracted by soaking in dichloromethane and ethyl acetate (3 × 250 mL; Merck, Darmstadt, Germany). The extracts were filtered through Whatman No. 1 filter paper (Whatman, Maidstone, UK) to discard plant residues, and the filtrates were concentrated under reduced pressure using a vacuum rotary evaporator (Buchi, Flawil, Switzerland). Then, the extracts yielded 4.6 g of crude ethyl acetate extract of *G. extensum* (EAGE) and 5.8 g of crude dichloromethane extract of *G. extensum* (DEGE), both obtained as green solid residues.

#### 2.3.2. Vernodalin Isolation

After obtaining EAGE, vernodalin (VD) was isolated by implementing the column chromatography technique. The extracts were separated into several fractions and subjected to the gradient elution system, starting from 100% CH_2_Cl_2_ to 40% MeOH:CH_2_Cl_2_, yielding eleven fractions designated GE1-GE11. Fraction GE6 (497 mg) was further purified with a gradient from 1% to 40% MeOH:CH_2_Cl_2_, affording subfractions GE6_1-GE6_9. Among these, GE6_6 (220 mg) was selected for further separation with a 1% MeOH:CH_2_Cl_2_ gradient, resulting in GE6_61-GE6_69. Subfraction GE6_62 (105.5 mg) underwent further separation with a gradient elution starting from 50% ethyl acetate in hexane (EtOAc:hexane) to 10% MeOH in EtOAc (MeOH:EtOAc), yielding GE6_621-GE6_627. Eventually, GE6_624 (54.5 mg) was separated using a gradient from 50% EtOAc:hexane to 100% EtOAc, affording VD (32.0 mg).

#### 2.3.3. Chemical Structure Validation and Purity Inspection

To validate the chemical structure of VD, ^1^H-NMR was implemented to observe signals of several fractions that were obtained from column chromatography. The spectra were recorded in CDCl_3_ on a 300 MHz spectrometer (Avance 300, Bruker, Rheinstetten, Germany) and compared with prior spectroscopic data from Chukwujekwu et al. [16]. After the chemical structure of VD was validated, the purity of VD was confirmed by HPLC which was implemented by the Office of Scientific Instrument and Testing (OSIT) at Prince of Songkla University (Hat Yai, Songkhla, Thailand), Prince of Songkla University. This protocol implemented isocratic elution with a mobile phase consisting of 25:75% *v*/*v* acetonitrile to deionized water, at a flow rate of 1.0 mL/min, an injection volume of 10 µL, and a total run time of 15 min.

### 2.4. Phytochemical Identification

To identify the phytochemical composition of both crude extracts, both crude extracts were submitted to OSIT, Prince of Songkla University, for GC-MS analysis. The crude extract was analyzed using a 7820A gas chromatograph coupled with a 5975C connected to a mass spectrometer (GC-MS) system (Agilent Technologies, Waldbronn, Germany). Chromatographic separation was performed on an HP-5MS capillary column composed of 5% phenyl methyl siloxane (30 m × 250 μm × 0.25 μm; Agilent Technologies). The oven temperature was initially set at 80 °C and held for 3 min, followed by a gradual increase at a rate of 5 °C/min until reaching 280 °C, where it was maintained for an additional 5 min, resulting in a total run time of 48 min. A sample volume of 1 μL was injected with a split ratio of 10:1. Helium was used as the carrier gas at a constant flow rate of 1 mL/min. Mass spectrometric detection was conducted using electron ionization (EI) at 70 eV in full-scan mode over an m/z range of 35–350. Only compounds representing more than 1% of the total peak area and exhibiting a database matching similarity above 85 were considered for further analysis.

### 2.5. Vernodalin Quantification

To quantify VD inside both *G. extensum* crude extracts, HPLC was implemented. The crude extracts were submitted to the OSIT, Prince of Songkla University. Agilent 1100 system (Agilent, Waldbronn, Germany) was equipped with Agilent ChemStation software B.04.02 (118) for GC, LC, LC/MSD, CE, and UV-Vis detection, along with an A/D Systems-Rev. 08 interface. A 20 µL aliquot of each sample solution was injected into the system. Separation was carried out on a Symmetry C8 analytical column (4.6 × 150 mm, 3 µm particle size) maintained at 30 °C, using a mobile phase consisting of acetonitrile (Merck Millipore, Burlington, VT, USA) and water. Eluted compounds were monitored at a wavelength of 254 nm. Prior to each run, the column was equilibrated for 6 min, and before analysis, it was conditioned with the mobile phase for at least 3 h to ensure system stability. A standard calibration curve was generated using serial dilutions of isolated VD at serial concentrations (Supplementary Material, Figure S1). The resulting data were plotted and presented as both graphical and tabular representations to determine the VD content in each extract.

### 2.6. Experimental Methods

#### 2.6.1. Cell Culture

Two cancer models with different origins were used: breast cancer cell lines (MDA-MB-231 cells) and ovarian cancer cell lines (A2780 cells). In addition, to determine the selectivity of all tested compounds, non-cancerous cell lines, L-929 (murine fibroblast cells), were used in this study. MDA-MB-231 (ATCC^®^ HTB-26) was obtained from the American Type Culture Collection (ATCC, Manassas, VA, USA), while A2780 (Addexbio^®^ C0017002) and L-929 (Cytion^®^ 400260) cells were purchased from AddexBio (San Diego, CA, USA) and Cytion (Eppelheim, Germany). A2780 cells were cultured in RPMI-1640 medium, while MDA-MB-231 and L-929 cells were cultured in Dulbecco’s Modified Eagle Medium (DMEM). All types of culture medium were supplemented with 10% fetal bovine serum (FBS), 1% penicillin–streptomycin, and 1% L-Glutamine. Cells were maintained at 37 °C in a humidified atmosphere containing 5% CO_2_ to ensure optimal viability and physiological relevance.

#### 2.6.2. MTT Assay

To evaluate the cytotoxic potential of VD and both *G. extensum* extracts, a standard MTT (3-(4,5-dimethylthiazol-2-yl)-2,5-diphenyltetrazolium bromide) colorimetric assay was performed by adopting our previous protocol [4]. Breast cancer cells (MDA-MB-231) and ovarian cancer cells (A2780) were seeded in 96-well plates (SPL Biosciences, Pocheon-si, Republic of Korea) at densities: 8 × 10^3^ cells/well for MDA-MB-231 and 1 × 10^4^ cells/well for A2780 cells. Then, the medium was replaced with fresh culture medium containing serial dilutions of VD at 1.73, 3.46, 6.94, 13.87, 27.75 μM or each extract at 5, 10, 20, 40, 80 μg/mL, incubated for 72 h. DOX and CIS served as positive controls at serial concentrations (0.625, 1.25, 2.5, 5, and 10 μM), while 0.5% *v*/*v* DMSO served as vehicle control. Untreated cells maintained in complete culture medium served as negative controls. Subsequently, MTT solution incubation was performed for 30 min and DMSO incubation for 30 min. Eventually, absorbance was measured at 570 nm and 650 nm using a Varioskan Lux Multimode Microplate Reader (Thermo Fisher Scientific, Waltham, MA, USA). Half-maximal inhibitory concentrations (IC_50_) were calculated to determine cytotoxic levels of tested compounds. Test concentrations used in subsequent experiments were expressed as fractions of the IC_50_ values determined from this assay.

#### 2.6.3. Clonogenic Assay

Due to MDA-MB-231 and A2780 cells responding to cytotoxic effects after being treated with VD, both cells were projected into further study. Firstly, a clonogenic (colony formation) assay was carried out to evaluate the long-term survival and proliferative capacity of cells after being exposed to VD and both extracts. The assay protocol was adapted from Mad-Adam and colleagues [17]. MDA-MB-231 and A2780 cell lines were seeded in 3.5 cm culture dishes at densities of 2 × 10^3^ and 1 × 10^3^ cells per dish, respectively. After overnight incubation at 37 °C to allow cell attachment, the cells were treated with serial concentrations of VD, DEGE, and EAGE within 72 h. DOX and CIS were assigned as positive controls for MDA-MB-231 and A2780 cells, respectively.

Following treatment, cells were gently washed and re-cultured in fresh complete medium within five days, with medium changes every two days. At the end of the incubation period, colonies were fixed with ice-cold 70% methanol. Fixed cells were stained with 2 mL of crystal violet solution, 5% for MDA-MB-231 and 0.5% for A2780, prepared in 25% methanol. After staining, excess dye was removed by rinsing the plates twice with distilled water, and plates were air-dried. Colonies consisting of at least 50 cells were counted under an Olympus CKX53 inverted microscope (Olympus, Hachioji, Japan). The percentage of colony formation was calculated using the following formula:

$$Colony\;Formation\;\left(\%\right)=\;\frac{Number\;of\;Colonies\;in\;treated\;group}{Number\;of\;Colonies\;in\;non-treated\;group}\times \;100$$

#### 2.6.4. Cell Cycle Analysis

To evaluate the potential of VD, DEGE, and EAGE to induce cell cycle arrest, flow cytometric analysis was conducted as previously described by Rattanaburee and colleagues [18]. MDA-MB-231 (breast cancer) and A2780 (ovarian cancer) cells were seeded into 12-well plates at a density of 2 × 10^5^ cells/well overnight. Cells were subsequently treated with the IC_50_ of VD, DEGE, or EAGE for 48 h. Cells were collected and centrifuged to obtain cell pellets. Cell pellets were fixed in 70% ice-cold ethanol and incubated for 3 h at 4 °C. Cells were stained with propidium iodide (PI) and incubated in the dark at room temperature. Finally, cell cycle distribution was analyzed using Muse^®^ Cell Analyzer (microcapillary-based fluorescence cytometry) (Merck, Darmstadt, Germany).

#### 2.6.5. Wound Healing Assay

To evaluate the anti-migratory potential of VD and crude extracts, a wound healing assay was performed using MDA-MB-231 cells. This protocol was adapted from the study by Tedasen and colleagues [19]. Cells were seeded at a density of 4 × 10^5^ cells/well in a 12-well plate and allowed to grow overnight. A scratch was introduced along the center of each well using a sterile 200 µL pipette tip to create a wound gap. The culture medium was replaced with fresh medium containing various concentrations of VD, DEGE, or EAGE. Untreated wells served as negative controls. Plates were incubated for 24 h, after which the wound closure was evaluated under an inverted microscope. Images of the wound area were captured at 0 and 24 h post-treatment. Cell migration was quantified by measuring the average wound width at time 0 (W0) and after 24 h (Wt). The migration rate was calculated using the following formula:

$$\%\;of\;Wound\;Closure=\left[\frac{\left(W0\;-\;Wt\right)}{W0}\right]\times 100$$

#### 2.6.6. Western Blot Analysis

To determine the protein levels of HSP90α and selected associated proteins of MDA-MB-231 and A2780 after exposure to the IC_50_ concentration of VD, Western blot analysis was conducted. DEGE, EAGE, and 17-AAG (an HSP90α inhibitor) were exposed to both cancer cells for 0, 24, and 48 h at a temperature of 37 °C. Then, cell pellets were extracted using ice-cold RIPA buffer (Thermo Fisher Scientific, Waltham, MA, USA). The collected protein concentrations were measured by the Bio-Rad Bradford protein assay dye (Bio-Rad Laboratories, Inc., San Fransicso, CA, USA). The weighed protein (15 µg) was separated by 12% SDS-PAGE and transferred onto nitrocellulose membranes (EMD Millipore, Darmstadt, Germany).

Membranes were blocked with 5% non-fat dry milk in 1× TBST for 1 h at room temperature and subsequently incubated with primary antibodies against HSP90α (1:500, rabbit mAb), SMYD3 (1:500, rabbit mAb), BRAF (1:500, rabbit mAb), MELK (1:500, rabbit mAb), Cyclin B1 (1:250, mouse mAb), MDR1/ABCB1 (1:500, rabbit mAb), and MMP-9 (1:500, rabbit mAb) for 2 h at room temperature. GAPDH (1:1000, rabbit mAb) was used as a loading control and incubated for 1 h. Then, membranes were washed three times with TBST and incubated with an HRP-conjugated secondary antibody (1:1500) at room temperature. Excess antibodies were removed with three washes in TBST. Protein bands were visualized using enhanced chemiluminescent (ECL) substrates (SuperSignal™ West Pico PLUS, West Dura, or Femto, Thermo Fisher Scientific) and imaged using a Bio-Rad ChemiDoc™ MP imaging system. Band intensities were quantified using Image Lab 6.0.1 software (Bio-Rad Laboratories, Inc., San Fransicso, CA, USA).

#### 2.6.7. Statistical Analysis

All experiments were independently performed at least twice. All data were presented as a mean ± standard deviation (SD). Statistical comparisons between treated and non-treated groups were conducted using an unpaired Student’s *t*-test in Microsoft Excel (Microsoft Corp., Redmond, WA, USA). Differences were considered statistically significant at * *p* < 0.05, ** *p* < 0.001, or *** *p* < 0.0001.

## 3. Results

### 3.1. Vernodalin Chemical Structural Validation

VD was obtained [32.0 mg, colorless viscous liquid; [*α*]_D_ +23.5 (*c* 0.78, CHCl_3_)]; ^1^H NMR (CDCl_3_) (*δ* ppm) (300 MHz): 6.74 (*brs*, 1H), 6.30 (*s*, 1H), 6.21 (*d*, *J* = 3.0 Hz, 1H), 5.97 (*s*, 1H), 5.96 (*brs*, 1H), 5.73 (*dd*, *J* = 10.8, 17.4 Hz, 1H), 5.65 (*d*, *J* = 3.0 Hz, 1H), 5.32 (*d*, *J* = 10.8 Hz, 1H), 5.27 (*d*, *J* = 17.4 Hz, 1H), 5.18 (*td*, *J* = 6.0, 10.5 Hz, 1H), 4.49 (*d*, *J* = 12.5 Hz, 1H), 4.35 (*s*, 2H), 4.27 (*d*, *J* = 12.5 Hz, 1H), 4.05 (*t*, *J* = 11.0 Hz, 1H), 3.03 (*d*, *J* = 12.0 Hz, 1H), 2.98 (*tt*, *J* = 2.7, 11.1 Hz, 1H), 2.22 (*dd*, *J* = 4.5, 14.1 Hz, 1H), 1.68 (*dd*, *J* = 10.2, 14.4 Hz, 1H).

### 3.2. Chemical Composition of EAGE and DEGE

GC-MS was performed to identify qualitatively and quantitatively volatile constituent profiles of the extracts. GC-MS analysis revealed distinct compound distributions in the DEGE and EAGE, pinpointing divergent bioactive constituent patterns. All identified compounds and the graphs for both extracts are presented in Supplementary Material in Tables S2 and S3 and Figure S6. GC-MS analysis revealed distinct phytochemical profiles between the extracts. The EAGE fraction was predominantly enriched in neophytadiene (18.29%), phytol (15.33%), phytol acetate (9.15%), phytyl acetate (6.01%), chondrillasterol (5.65%), and phytyl palmitate (5.49%). In contrast, DEGE exhibited a markedly different composition, with phytyl palmitate (12.69%) as the most abundant constituent, followed by neophytadiene (11.81%), phytol (7.66%), chondrillasterol (7.53%), tetratriacontane (7.44%), and squalene (6.72%). These compositional differences suggest that the extraction solvent substantially influences the relative abundance of bioactive compounds such as terpenoid, sterol, and long-chain hydrocarbon constituents.

### 3.3. Isolation, Purity Inspection, and Quantitative Determination of VD in EAGE and DEGE

**Figure 1:**
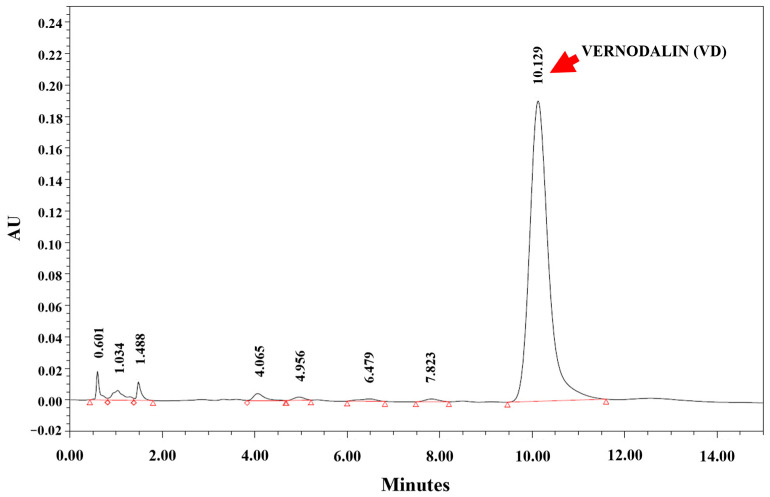
HPLC chromatogram of VD after purification by column chromatography. A major peak corresponding to VD was pointed by red arrow and it confirms the high purity of the isolated compound (>92%).

**Figure 2:**
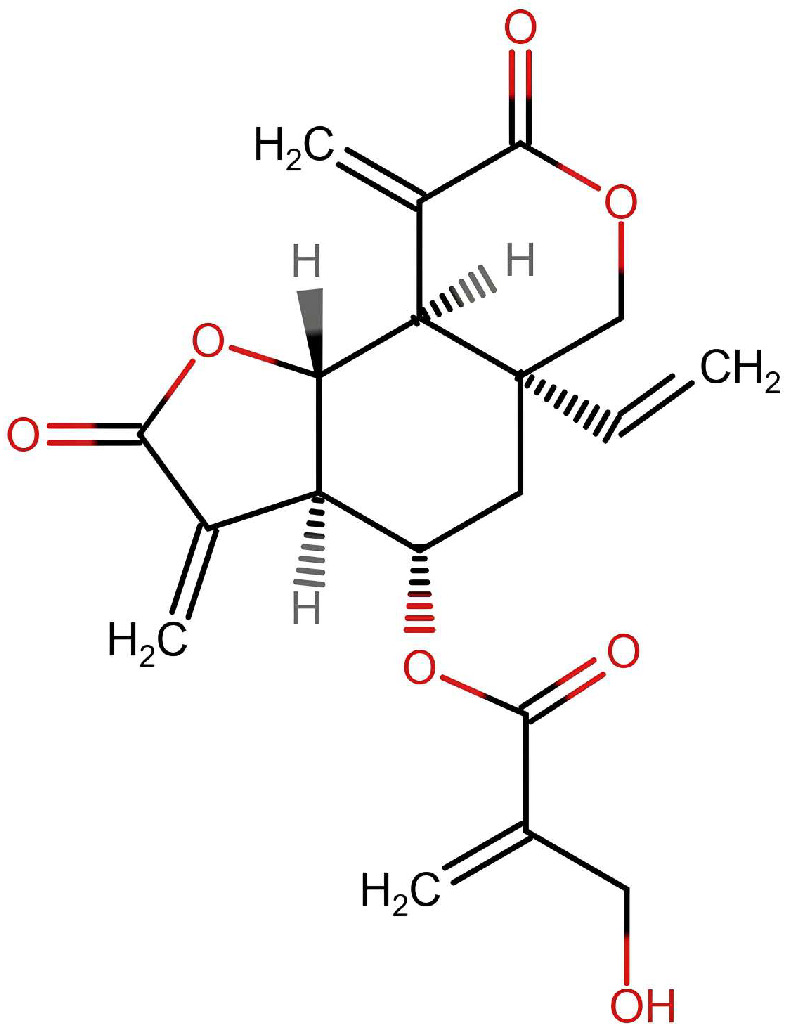
VD chemical structure drawn in RCSB Chemical Sketch. Red-colored atoms and bonds denote oxygen atoms and oxygen-containing functional groups present in the molecule. (https://www.rcsb.org/search/chemical, accessed on 6 May 2026).

Vernodalin was isolated from the crude ethyl acetate extract, and its structure was elucidated based on spectroscopic data (Supplementary Material in Figures S1–S4 and Table S1) and compared with the literature [16]. Subsequently, the purity of VD isolated from EAGE was determined to be greater than 92%, as confirmed by HPLC analysis (Figure 1). The VD content in both EAGE and DEGE was then quantitatively determined using HPLC. We also calibrated the HPLC results using isolated VD and obtained the correlation coefficient (*r*^2^) at 0.99936 (see Supplementary Figure S5), which is acceptable for HPLC calibration [20]. VD was found inside DEGE with a concentration of 50.86 ± 1.52 mg/g and 11.14 ± 0.18 mg/g inside EAGE, see Supplementary Material in Figure S7. The chemical structure of VD was depicted in Figure 2.

### 3.4. Cytotoxicity Evaluation

Cells were treated with increasing concentrations of VD, DEGE, EAGE, DOX, CIS, and 17-AAG for 72 h, after which cell viability was assessed using the MTT assay. As presented in Table 1, VD, DEGE, and EAGE produced dose-dependent cytotoxic effects in both cancer cell lines. VD, DEGE, 17-AAG, and DOX exhibited the greatest inhibitory activity against A2780 ovarian cancer cells, whereas EAGE showed the highest cytotoxic effect on MDA-MB-231 breast cancer cells. In contrast, VD, DEGE, and EAGE demonstrated comparatively low cytotoxicity towards normal L-929 fibroblasts. Notably, the conventional chemotherapeutic agents DOX and CIS displayed pronounced toxicity in L-929 cells.

### 3.5. Colony Formation Reduction

**Figure 3:**
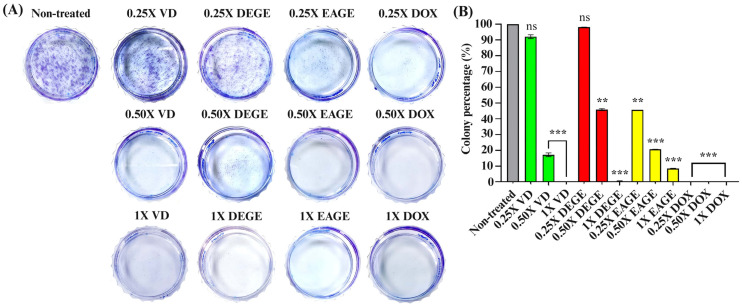
Inhibitory effects of VD, DEGE, and EAGE on colony formation in MDA-MB-231 breast cancer cells. Following 72 h exposure to increasing concentrations of VD, DEGE, EAGE, or DOX, cells were allowed to recover for 5 days in drug-free medium. Colony formation was visualized by crystal violet staining (**A**) and quantitatively expressed as the percentage of colonies relative to the non-treated control (**B**). Data represents the mean ± SD from two independent experiments. ns: non-statistically significant. ** *p* < 0.01, and *** *p* < 0.001 denote statistically significant differences compared with the non-treated group.

**Figure 4:**
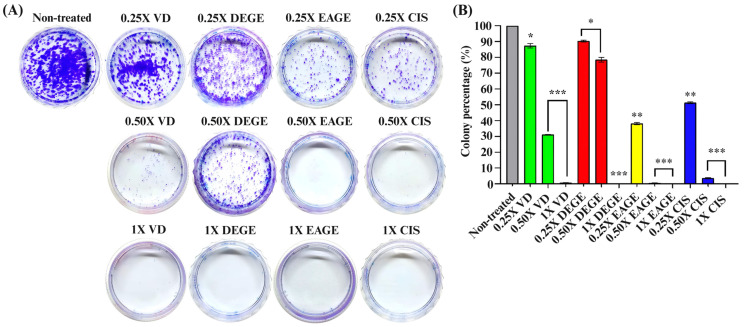
Inhibitory effects of VD, DEGE, and EAGE on colony formation in A2780 ovarian cancer cells. Following 72 h exposure to increasing concentrations of VD, DEGE, EAGE, or CIS, cells were allowed to recover for 5 days in drug-free medium. Colony formation was visualized by crystal violet staining (**A**) and quantitatively expressed as the percentage of colonies relative to the non-treated control (**B**). Data represents the mean ± SD from two independent experiments. * *p* < 0.05, ** *p* < 0.01, and *** *p* < 0.001 denote statistically significant differences compared with the non-treated group.

The clonogenic assay was conducted to evaluate the effects of serially diluted concentrations of VD, DEGE, and EAGE on colony formation of MDA-MB-231 and A2780 cells. DOX and CIS, used as positive controls, reduced colony formation by approximately 90–100%. All treatments produced dose-dependent inhibition of colony formation in both cell lines. Notably, treatment with 0.5× IC_50_ concentrations of VD and EAGE significantly reduced colony numbers to less than 50% of those observed in non-treated cells. Moreover, EAGE exhibited a stronger inhibitory effect on colony formation than DEGE. At 0.5× IC_50_, DEGE inhibited colony formation more effectively in MDA-MB-231 cells than in A2780 cells (Figure 3 and Figure 4). Overall, these results indicate that VD and the plant extracts suppressed colony formation in breast and ovarian cancer cells.

### 3.6. Cell Cycle Distribution

**Figure 5:**
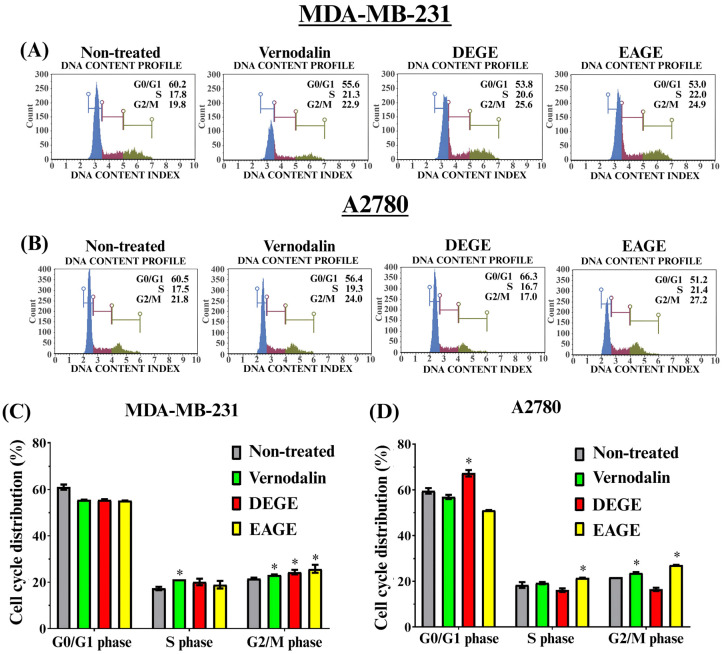
Modulation of cell cycle progression by VD, DEGE, and EAGE in breast and ovarian cancer cells. (**A**,**B**) Representative cell cycle phase distributions obtained using the Muse^®^ Cell Analyzer after 48 h treatment with VD, DEGE, or EAGE are shown for MDA-MB-231 cells and A2780 cells, with non-treated cells included as controls. (**C**,**D**) Quantitative analysis of the proportion of cells in each cell cycle phase was presented for MDA-MB-231 and A2780. Data are expressed as mean ± SD. * *p* < 0.05 indicates statistically significant differences compared with the non-treated control.

The effects of VD, DEGE, and EAGE on cell cycle distribution were examined in two cancer cell lines of distinct origin. In MDA-MB-231 cells, treatment with VD, DEGE, and EAGE resulted in a statistically significant accumulation of cells in the G2/M phase compared with the non-treated control (Figure 5A,D; *p* < 0.05). In A2780 cells, VD and EAGE predominantly induced G2/M phase arrest, whereas DEGE caused a significant increase in the G0/G1 cell population (Figure 5B,D; *p* < 0.05 versus non-treated control). Overall, these findings indicate that VD and the plant extracts significantly alter cell cycle distribution in breast and ovarian cancer cells.

### 3.7. Wound Healing Evaluation

**Figure 6:**
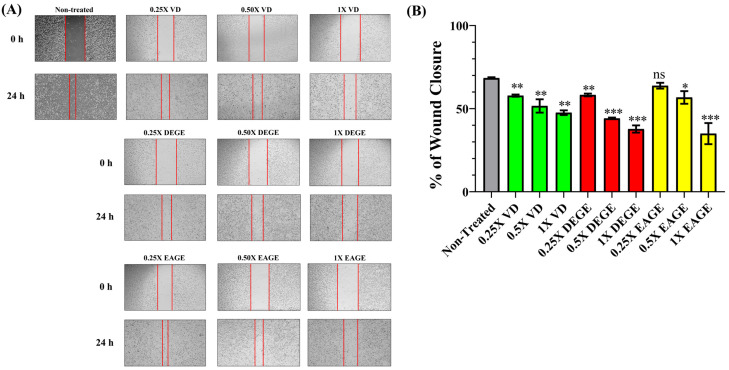
Wound healing assay of MDA-MB-231 cells after 24 h treatment with VD, DEGE, or EAGE at 0.25× IC_50_, 0.5× IC_50_, and 1× IC_50_. (**A**) Representative images of wound closure captured using an inverted microscope (×200 magnification). (**B**) Quantitative analysis of wound closure in MDA-MB-231 cells. Data are presented as mean ± SD from two independent experiments. ns: non-statistically significant. * *p* < 0.05, ** *p* < 0.01, and *** *p* < 0.001 indicate statistically significant differences compared with the non-treated control.

Cell migratory behavior was examined in MDA-MB-231 cells using a wound healing assay. Following scratch formation, cells were treated with VD, DEGE, or EAGE, and wound closure was monitored over a 24 h period. As shown in Figure 6, treatment with VD, DEGE, or EAGE resulted in a statistically significant delay in wound closure in a concentration-dependent manner compared with non-treated controls (*p* < 0.05). These findings suggest that VD and the plant extracts are associated with altered wound closure behavior in MDA-MB-231 cells.

### 3.8. Protein Expression Levels of HSP90α-Associated Proteins in MDA-MB-231 and A2780 Cells

**Figure 7:**
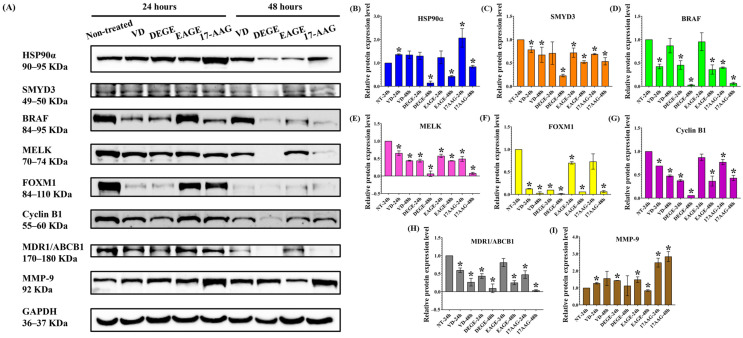
Effects of VD and *G. extensum* extracts on HSP90α and downstream protein levels in breast cancer cells (MDA-MB-231). (**A**) MDA-MB-231 cells were treated with VD, DEGE, EAGE, or 17-AAG for 24 and 48 h. Protein levels of HSP90α and downstream proteins (SMYD3, BRAF, MELK, FOXM1, Cyclin B1, MDR1/ABCB1, and MMP-9) were determined by Western Blot analysis. (**B**–**I**) Densitometric analysis of immunoreactive bands was performed, and protein levels were normalized to GAPDH as a loading control. All data are presented as the mean ± SD from two independent experiments. * *p* < 0.05 compared with the non-treated control group.

Western blot analysis was performed to determine the protein levels of HSP90α and selected downstream proteins associated with HSP90α signaling in breast cancer (MDA-MB-231) and ovarian cancer (A2780) cells following treatment with VD, DEGE, EAGE, or 17-AAG, a well-characterized HSP90α inhibitor used as a positive control [21,22]. Cells were incubated for 24 and 48 h, and protein levels were compared with those of non-treated control cells. At 48 h, treatment with DEGE, EAGE, and 17-AAG decreased HSP90α protein levels and was accompanied by reduced levels of SMYD3, MELK, FOXM1, Cyclin B1, and MDR1/ABCB1. In contrast, VD did not reduce HSP90α protein levels but decreased the expression of FOXM1, MELK, Cyclin B1, and MDR1/ABCB1. DEGE produced the greatest overall reduction in protein levels among the tested treatments at 48 h, with the exception of MMP-9. Moreover, levels of MMP-9 protein were increased following treatment with VD, DEGE, and 17-AAG, whereas EAGE reduced MMP-9 protein levels at 48 h (Figure 7).

**Figure 8:**
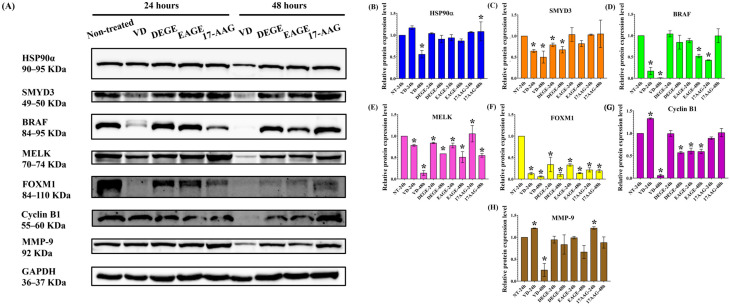
Effects of VD and *G. extensum* extracts on HSP90α and downstream protein levels in ovarian cancer cells (A2780). (**A**) A2780 cells were treated with VD, DEGE, EAGE, or 17-AAG for 24 and 48 h. Protein levels of HSP90α and downstream proteins (SMYD3, BRAF, MELK, FOXM1, Cyclin B1, and MMP-9) were determined by Western Blot analysis. (**B**–**H**) Densitometric analysis of immunoreactive bands was performed, and protein levels were normalized to GAPDH as a loading control. All data are presented as the mean ± SD from two independent experiments. * *p* < 0.05 compared with the non-treated control group.

In A2780 cells, treatment with VD alone reduced HSP90α protein levels and its associated oncogenic proteins, including SMYD3, BRAF, MELK, FOXM1, Cyclin B1, and MMP-9. The known HSP90α inhibitor 17-AAG decreased the levels of BRAF, MELK, and FOXM1. In contrast, DEGE and EAGE exhibited differential suppressive effects on SMYD3 and BRAF. Notably, the inhibitory effects on all proteins were more pronounced at 48 h, with VD demonstrating the strongest overall suppression (Figure 8). Overall, these findings suggest that VD exerts stronger inhibitory effects on HSP90α-associated oncogenic signaling pathways than 17-AAG in A2780 cells.

## 4. Discussion

*G. extensum* (Bitter Leaf Tree) has emerged as a promising medicinal plant with notable anticancer properties. While our previous studies demonstrated that DEGE and EAGE of *G. extensum* exert potent cytotoxic effects against several cancer cell lines [4], the molecular mechanisms underlying these activities remained largely unexplored. Therefore, the present study investigated the anticancer effects and associated molecular responses of VD, a major bioactive constituent of *G. extensum*, together with DEGE and EAGE. Consistent with our previous findings, GC-MS analyses identified phytol, neophytadiene, and palmitate derivatives among the major constituents of the extracts. In the present study, DEGE was enriched in phytyl palmitate, neophytadiene, phytol, and chondrillasterol, whereas EAGE contained higher levels of neophytadiene, phytol acetate, and phytyl acetate. These differences in phytochemical composition may partly account for the distinct anticancer activities and molecular regulatory effects observed between the two crude extracts.

The distinct biological activities observed between DEGE and EAGE are likely attributable to the complex interactions among multiple phytochemicals rather than the action of a single constituent. Numerous compounds identified in both extracts have previously been reported to suppress proliferation, induce apoptosis, and inhibit migration through diverse molecular mechanisms [23,24,25,26,27,28]. Consequently, the overall anticancer effects of the crude extracts may arise from additive, synergistic, or even antagonistic interactions among their constituents. Such phytochemical complexity may explain the differences in cytotoxicity and molecular responses observed between DEGE and EAGE. Further studies are required to define the contribution of individual compounds and their interactions to the overall biological activity of the extracts.

The selectivity of VD, DEGE, and EAGE was further evaluated using non-cancerous L-929 cells as a representative normal cell model. L-929 cells, a mouse fibroblast cell line recommended by international standards for biological safety assessment of medical devices [29], are widely used in cytotoxicity studies because of their reproducibility and high sensitivity to toxic stimuli [30]. The results demonstrated that VD, DEGE, and EAGE exhibited greater cytotoxic effects toward cancer cells than toward L-929 cells, suggesting a degree of selective anticancer activity. However, L-929 cells do not fully recapitulate the physiological characteristics of normal human breast or ovarian tissues; therefore, these findings should be interpreted as preliminary evidence of selectivity and safety. Further studies using relevant normal human cell models are required to validate the therapeutic window and tissue-specific safety profiles of these treatments.

Our previous computational study identified HSP90α, together with JAK3, IMPDH2, COX-2, AKR1B1, Cyclin B1, MetAP2, and CDC25A, as potential molecular targets of VD [9]. HSP90α plays a central role in regulating multiple oncogenic signaling networks [31,32]. HSP90α and selected downstream proteins were included in the present investigation to explore molecular changes associated with the anticancer effects of VD and *G. extensum* extracts. Furthermore, several major phytochemicals identified in DEGE and EAGE have previously been reported to influence pathways involved in cell proliferation, survival, and migration [23,24,28,33], supporting the relevance of examining these protein networks.

The present findings suggest that VD exerts anticancer effects through context-dependent molecular responses that vary between cancer cell types. While VD broadly suppressed proteins involved in proliferation, survival, and migration, differences in the extent of protein modulation were observed between MDA-MB-231 and A2780 cells. Moreover, the ability of VD to inhibit migration despite limited changes in certain migration-associated proteins suggests the involvement of additional signaling pathways regulating cell motility [34]. These differential responses may reflect intrinsic differences in signaling networks and adaptive mechanisms operating within each cancer subtype. Previous studies have shown that VD induces ROS accumulation and suppresses NF-κB and FAK signaling pathways [8,35], which play pivotal roles in cancer cell proliferation, survival, migration, and stress adaptation [36,37,38,39]. The limited reduction of HSP90α and MMP-9 in MDA-MB-231 cells may be associated with the highly adaptive nature of TNBC cells, which exhibit elevated NF-κB activity and enhanced stress-response mechanisms [40,41]. In addition, MDA-MB-231 cells express high levels of PITPNM3, a regulator of Pyk2/FAK/Src signaling associated with MMP-9 expression and metastatic behavior [42,43,44,45]. Hence, although VD effectively suppressed several oncogenic proteins in both cancer cell lines, the extent of HSP90α and MMP-9 modulation appears to be influenced by the intrinsic signaling characteristics of each cancer subtype.

In contrast to VD, the crude extracts exhibited a more pronounced effect on MMP-9 expression in MDA-MB-231 cells, suggesting that phytochemicals present in DEGE and EAGE may contribute to the regulation of migratory behavior. This observation is consistent with previous reports demonstrating that neophytadiene, palmitate derivatives, and phytol can suppress the invasive and migratory capacities of aggressive cancer cells [24,33,46,47]. Nevertheless, the interpretation of the migration data should be made with caution, as the scratch-wound assay is subject to several limitations, including interference from cell proliferation, release of intracellular content following mechanical injury, and limited reproducibility [48]. Thus, additional migration assays are required to further validate the antimigratory effects of VD and *G. extensum* extracts.

Comparison of VD and 17-AAG revealed both shared and distinct molecular responses. In both cancer cell lines, the two compounds reduced the expression of several proteins associated with cancer cell proliferation and migration, including BRAF, MELK, FOXM1, and Cyclin B1, suggesting convergence on signaling networks that support tumor progression. Nevertheless, differences in protein modulation were also observed. In MDA-MB-231 cells, 17-AAG produced a greater reduction in HSP90α expression than VD, whereas VD more effectively suppressed HSP90α in A2780 cells. Given that 17-AAG is a well-established HSP90 inhibitor that primarily targets HSP90 ATPase activity rather than directly suppressing HSP90 expression [49], these findings suggest that VD and 17-AAG may influence overlapping oncogenic pathways despite exhibiting distinct patterns of protein regulation. Notably, the ability of VD to suppress multiple proteins involved in proliferation, survival, and migration to an extent comparable to 17-AAG further supports its broad anticancer activity across different cancer cell types.

The molecular responses induced by DEGE and EAGE may be partly attributable to the biological activities of their major phytochemical constituents. Phytol has been reported to induce ROS accumulation and apoptosis through caspase-dependent pathways while suppressing signaling pathways associated with cell proliferation and survival, including MAPK- and PI3K/AKT-related networks [25]. Likewise, neophytadiene-rich extracts have demonstrated antiproliferative and antimigratory activities accompanied by modulation of PI3K/AKT, MAPK, and NF-κB signaling [23,50]. In addition, palmitate derivatives have been associated with growth inhibition, apoptosis induction, and reduced invasiveness through regulation of PI3K/AKT, JAK2/STAT3, p53, BCL-2, and NF-κB pathways [26,33,47,51], whereas phytol acetate, phytyl acetate, and chondrillasterol have also shown cytotoxic activities in various cancer models [27,28,52]. Collectively, these findings suggest that the molecular effects of DEGE and EAGE are likely mediated through the combined actions of multiple phytochemicals capable of modulating interconnected signaling pathways involved in cancer cell proliferation, survival, and migration. Such phytochemical complexity may contribute to the broad protein regulatory effects observed in the present study.

**Figure 9:**
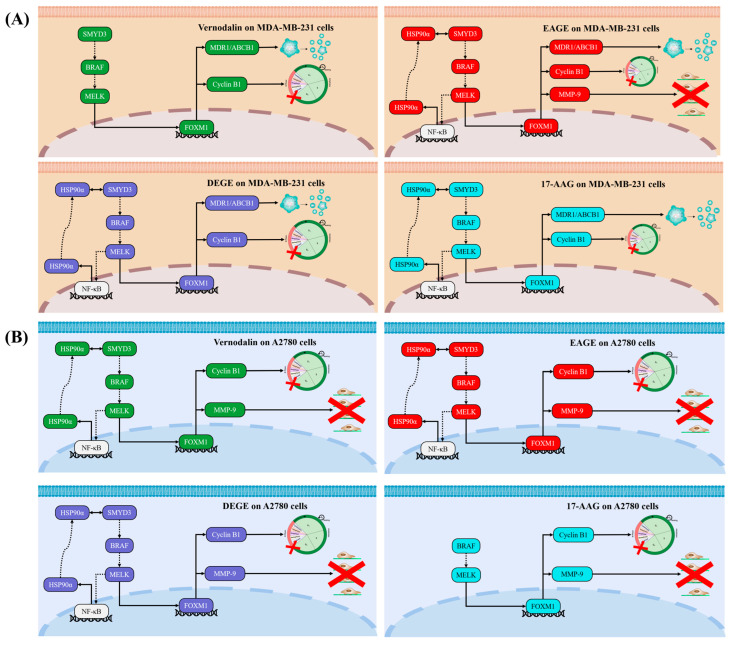
Proposed molecular mechanisms of VD and comparison with DEGE, EAGE, and 17-AAG on HSP90α-associated signaling pathways in (**A**) MDA-MB-231 and (**B**) A2780 cells. Several proteins involved in HSP90α-regulated chaperone signaling, including SMYD3, BRAF, MELK, and FOXM1, were downregulated following treatment. Notably, VD and the crude extracts modulated distinct protein expression profiles in the two cancer cell lines, suggesting that VD is not the sole contributor to the anticancer activity of the extracts and that HSP90α-associated signaling is regulated in a cell-dependent manner. SMYD3 downregulation may contribute to reduced BRAF expression through disruption of MAP3K2-mediated RAS signaling [53]. In turn, BRAF suppression may indirectly decrease MELK expression via inhibition of the RAF–MEK–ERK–E2F1 signaling axis [54]. Reduced MELK levels may subsequently impair oncogenic regulators such as FOXM1 [55] and NF-κB [54], leading to decreased expression of proteins associated with cell proliferation (Cyclin B1), cell survival and drug resistance (MDR1/ABCB1), and cell migration (MMP-9). Owing to NF-κB can transcriptionally regulate HSP90α expression [41], its activation may contribute to elevated HSP90α levels in cancer cells. Dashed lines indicate indirect regulatory relationships. Colored shapes denote proteins downregulated by VD (green), DEGE (blue), EAGE (red), and the HSP90 inhibitor 17-AAG (cyan).

Our findings demonstrate that VD and *G. extensum* crude extracts exhibit significant in vitro anticancer activity and are associated with alterations in several proteins linked to cancer progression, including components of HSP90α-related signaling networks. The distinct molecular responses observed between VD and the crude extracts suggest that multiple phytochemicals contribute to the overall anticancer activity of *G. extensum*. Although these findings provide valuable insight into potential molecular mechanisms, the direct molecular targets of VD and the precise relationships among the affected signaling pathways remain to be clarified. Future studies should include target-validation experiments, direct binding analyses, and in vivo investigations to further evaluate the therapeutic potential, safety, and bioavailability of VD and *G. extensum* extracts. A schematic overview of the proposed molecular responses associated with VD and crude extract treatment is presented in Figure 9.

## 5. Conclusions

DEGE and EAGE from *G. extensum* exhibited distinct phytochemical profiles and anticancer activities in MDA-MB-231 and A2780 cells. VD, a minor constituent of both extracts, demonstrated potent inhibitory effects on colony formation, cell cycle progression, and cell migration, accompanied by alterations in proteins associated with cancer cell proliferation, survival, and migration. The distinct molecular responses observed between VD and the crude extracts suggest that the anticancer effects of *G. extensum* may arise from the combined actions of multiple phytochemicals. Overall, these findings support the in vitro anticancer potential of VD and *G. extensum* extracts, while further mechanistic and in vivo studies are needed to clarify their therapeutic relevance.

## Figures and Tables

**Table 1:** Cytotoxic effects of VD, DEGE, EAGE, DOX, CIS and 17-AAG on MDA-MB-231, A2780, and L-929 cells.

Treatment	IC_50_ (mean ± SD)		
	MDA-MB-231	A2780	L-929
VD ^a^	5.69 ± 0.12	3.77 ± 0.60	9.75 ± 0.31
DEGE ^b^	15.70 ± 0.98	13.45 ± 0.39	29.51 ± 8.33
EAGE ^b^	11.71 ± 0.16	16.30 ± 1.19	27.03 ± 9.90
DOX ^a^	1.27 ± 0.48	0.67 ± 0.06	0.86 ± 0.39
CIS ^a^	24.65 ± 0.94	19.41 ± 0.93	9.97 ± 1.34
17-AAG ^a^	3.70 ± 0.35	0.006 ± 0.0007	0.49 ± 0.09

^a^ IC_50_ defined as µM; ^b^ IC_50_ defined as µg/mL.

## Data Availability

The original data generated and analyzed in this study are available within the article. Further inquiries can be directed to the corresponding author.

## Supplementary Materials

The following supporting information can be downloaded at: https://www.mdpi.com/article/10.3390/biomedicines14061331/s1, Figure S1: ^1^H-NMR Spectrum of VD (300 MHz, CDCl_3_); Figure S2: ^13^C NMR spectrum of VD (75 MHz, CDCl_3_); Figure S3: HMQC spectrum of VD; Figure S4: HMBC spectrum of VD; Figure S5: HPLC chromatogram of VD standard; Figure S6: GC-MS chromatograms and relative component peak areas; Figure S7: HPLC chromatograms; Table S1: NMR data comparison with literature; Table S2: Phytochemical constituents identified in EAGE using GC-MS; Table S3: Phytochemical constituents identified in DEGE using GC-MS.

## Abbreviations

The following abbreviations are used in this manuscript:

17-AAG	17-N-allylamino-17-demethoxygeldanamycin
AKR1B1	Aldo-Keto Reductase Family 1 Member B1
BCL2	B-Cell Lymphoma 2
BRAF	B-Rapidly Accelerated Fibrosarcoma
CDC25A	Cell Division Cycle 25A
CIS	Cisplatin
DEGE	Dichloromethane crude extract of *Gymnanthemum extensum*
DOX	Doxorubicin
E2F1	E2F Transcription Factor 1
EAGE	Ethyl acetate crude extract of *Gymnanthemum extensum*
ECL	Enhanced chemiluminescent
ERK	Extracellular Signal-Regulated Kinase
FAK	Focal Adhesion Kinase
FOXM1	Forkhead box M1
GAPDH	Glyceraldehyde-3-Phosphate Dehydrogenase
GC-MS	Gas Chromatography-Mass Spectrometry
HPLC	High-Performance Liquid Chromatography
HSP90α	Heat Shock Protein 90 Alpha
IC_50_	Half-maximum inhibitory concentration
JAK2	Janus Kinase 2
MAP3K2	Mitogen-Activated Protein Kinase Kinase Kinase 2
MAPK	Mitogen-Activated Protein Kinase
MCF-7	Michigan Cancer Foundation-7
MDA-MB-231	MD Anderson Metastatic Breast-231
MDR1/ABCB1	Multidrug Resistance 1/ATP-Binding Cassette Subfamily B member 1
MEK	Mitogen-Activated Protein Kinase Kinase
MELK	Maternal Embryonic Leucine Zipper Kinase
MetAP2	Methionine Aminopeptidase 2
MMP-9	Matrix Metalloproteinase 9
MTT	3-(4,5-dimethylthiazol-2-yl)-2,5-diphenyltetrazolium bromide
NF-κB	Nuclear Factor kappa-light-chain-enhancer of activated B cells
NMR	Nuclear Magnetic Resonance
PI3K	Phosphoinositide 3-kinase
PITPNM3	Phosphatidylinositol Transfer Protein Membrane-Associated 3
RT	Retention Time
TNBC	Triple-Negative Breast Cancer
SD	Standard Deviation
SMYD3	Set and Mynd domain-containing Protein 3
STAT3	Signal Transducer and Activator of Transcription 3
VD	Vernodalin
