# Supplementary material for: Vernodalin and Gymnanthemum extensum Crude Extracts Exhibit In Vitro Anticancer Activity with Differential Regulation of Cancer-Associated Signaling Proteins in Breast and Ovarian Cancer Cells
Source: Biomedicines. 2026 Jun 11;14(6):1331. doi: 10.3390/biomedicines14061331 (PMC13297053; doi:10.3390/biomedicines14061331)
Supplement: Supplementary file 1 [file biomedicines-14-01331-s001.zip › biomedicines-4284145-supplementary.pdf]

Supplementary Figure S1. ^{1}H-NMR Spectrum of VD (300 MHz, $CDCl_3$)

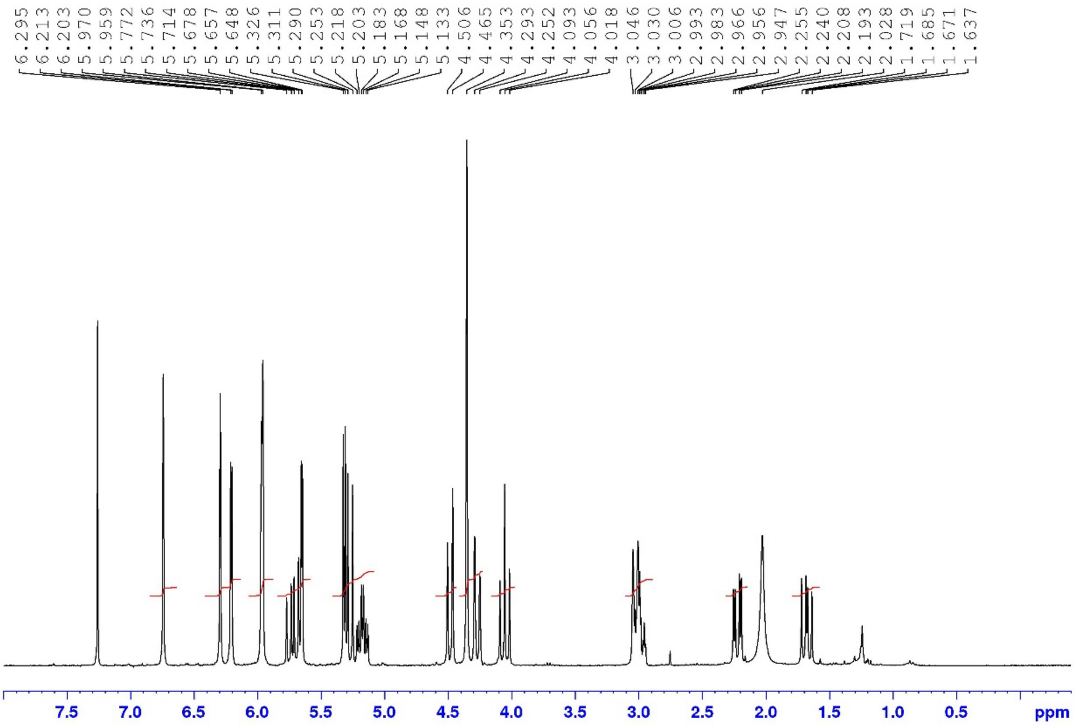

Supplementary Figure S2. ^{13}C NMR spectrum of VD (75 MHz, $CDCl_3$)

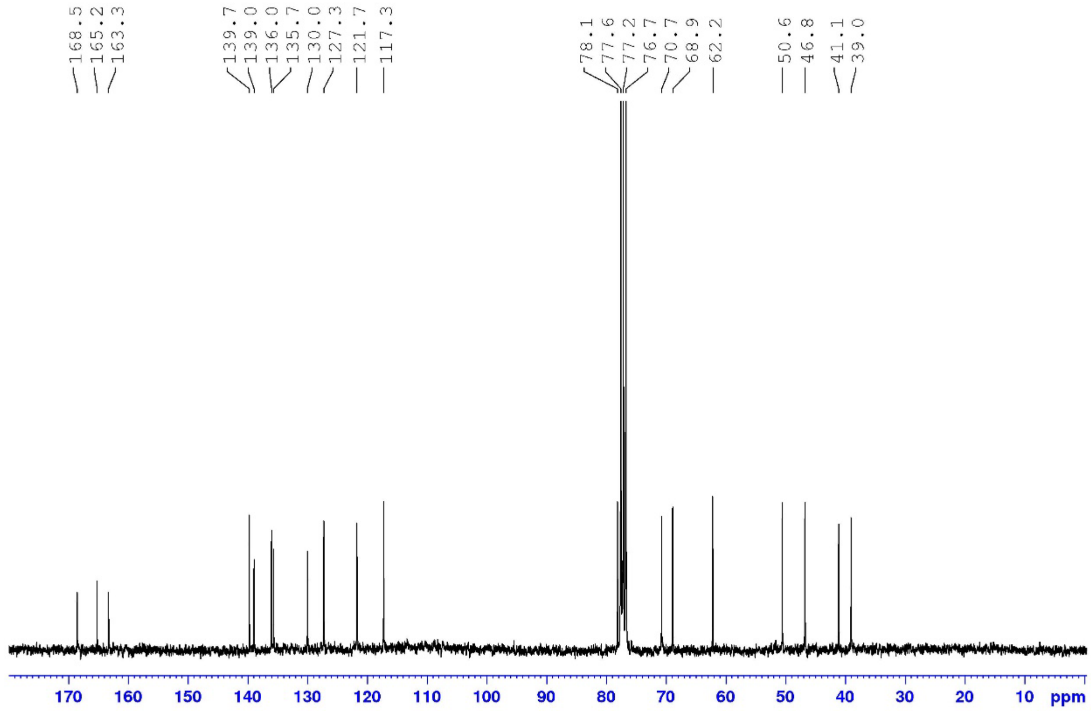

Supplementary Figure S3. HMQC spectrum of VD

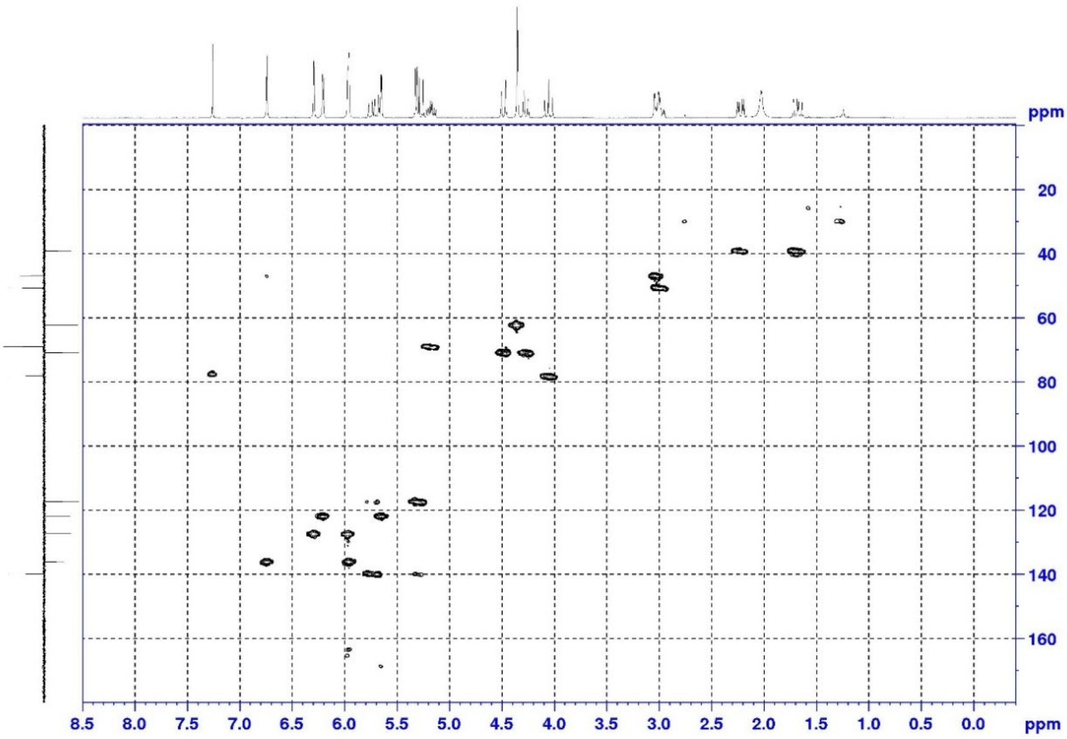

Supplementary Figure S4. HMBC spectrum of VD

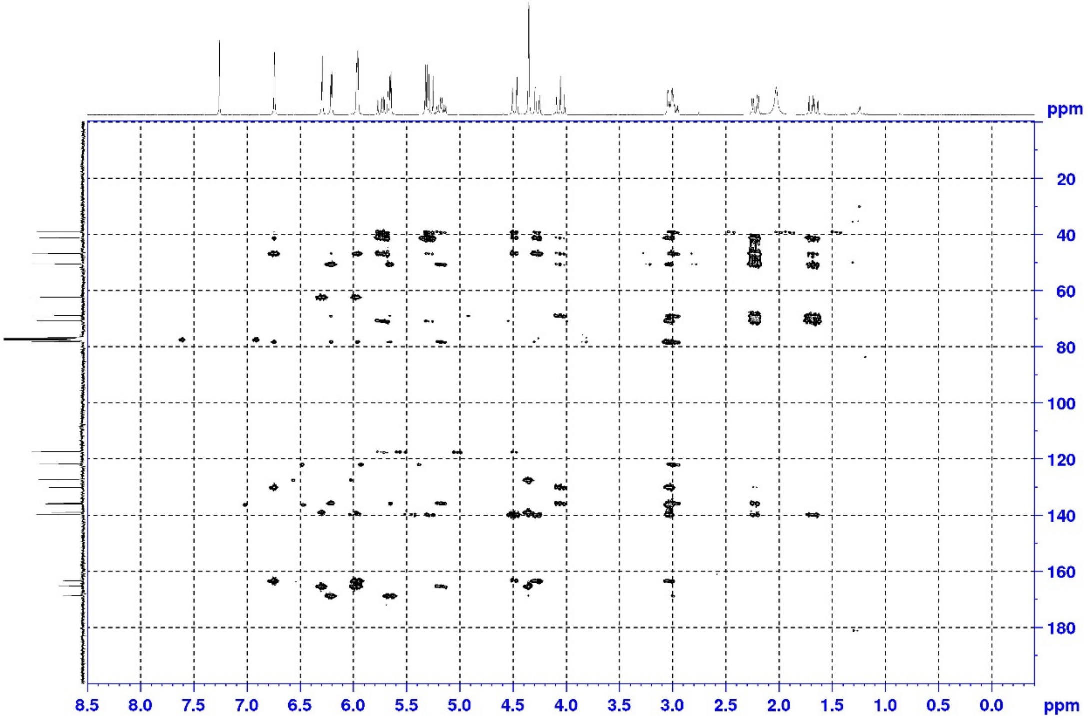

Supplementary Table S1. NMR data comparison with literature

position	VD ($CDCl_3$)		Vernodalin ($CDCl_3$+CD_3OD)	
	δ_H (*J*, in Hz)	δ_C, type	δ_H (*J*, in Hz)	δ_C, type
1	5.73 (*dd*,10.8,17.4)	139.8 (CH)	5.59 (*dd*,11.1, 17.5)	139.5 (CH)
2	5.32 (*d*, 10.8)<br>5.27 (*d*, 17.4)	117.3 (CH_2)	5.13 (*d*, 11.1)<br>5.12 (*d*, 17.5)	116.4 (CH_2)
3	-	163.3 (C)	-	163.7 (C)
4	-	130.0 (C)	-	129.9 (C)
5	3.03 (*d*, 12.0)	46.8 (CH)	2.93 (*d*, 11.1)	46.0 (CH)
6	4.05 (*t*, 11.1)	78.1 (CH_2)	4.01 (*t*, 11.5)	77.8 (CH_2)
7	2.98 (*tt*, 2.7, 11.1)	50.5 (CH)	2.89 (*tt*, 3.0, 11.0)	49.9 (CH)
8	5.18 (*td*, 6.0, 10.5)	68.9 (CH)	5.04 (*td*, 4.8, 10.5)	68.3 (CH)
9	2.22 (*dd*, 4.5, 14.1)<br>1.68 (*dd*, 10.2, 14.4)	39.0 (CH_2)	2.05 (*dd*, 4.6, 14.2<br>1.54 (*dd*, 10.3, 14.2)	38.4 (CH_2)
10	-	41.1 (C)	-	40.4 (C)
11	-	135.7 (C)	-	135.3 (C)
12	-	168.6 (C)	-	168.8 (C)
13	6.21 (*d*, 3.0)<br>5.65 (*d*, 3.0)	121.8 (CH_2)	6.01 (*d*, 3.0)<br>5.51 (*d*, 3.0)	121.0 (CH_2)
14	4.49 (*d*,12.5)<br>4.27 (*d*, 12.5)	70.7 (CH_2)	4.43 (*d*, 12.9)<br>4.12 (*d*, 12.9)	70.4 (CH_2)
15	6.74 (*s*)<br>5.96 (*s*)	136.0 (CH_2)	6.54 (*s*)<br>5.79 (s)	135.4 (CH_2)
1′	-	165.2 (C)	-	164.8 (C)
2′	-	139.0 (C)	-	139.1 (C)
3′	4.35 (*s*)	62.2 (CH_2)	4.14 (*s*)	60.8 (CH_2)
4′	6.30 (*s*)<br>5.97 (*s*)	127.3 (CH_2)	6.13 (*brs*)<br>5.82 (*brs*)	126.0 (CH_2)

Ref. [16] Chukwujekwu JC, Lategan CA, Smith PJ, Van Heerden FR, Van Staden J. Antiplasmodial and cytotoxic activity of isolated sesquiterpene lactones from the acetone leaf extract of *Vernonia colorata*. *South Afr J Bot*. **2009**, *75*, 176–179.

Supplementary Table S2. Phytochemical constituents identified in EAGE using GC-MS

RT (min)	Identified compound	Formula	Molecular mass (g/mol)	Area (%)	Compound nature	Match factor
**18.2824**	Copaene	$C_{15}H_{24}$	204.35	0.15	Hydrocarbon	91.6
**19.3896**	Caryophyllene	$C_{15}H_{24}$	204.35	0.37	Terpene	96.2
**22.0372**	Dihydroactinolide	$C_{11}H_{16}O_2$	180.24	0.68	Benzofuranone	95.6
**23.3637**	Caryophylene oxide	$C_{15}H_{24}O$	220.35	0.23	Terpene	85.1
**27.4127**	Loliolide	$C_{11}H_{16}O_3$	196.24	1.20	Benzofuranone	96.7
**28.8996**	Phytene-2	$C_{20}H_{40}$	280.50	1.29	Hydrocarbon	90.7
**29.0066**	Neophytadiene	$C_{20}H_{38}$	278.50	18.29	Terpene	97.1
**29.4933**	Phytol, acetate	$C_{22}H_{42}O_2$	338.60	9.15	Terpenoid	95.7
**32.0233**	Hexadecanoic acid, ethyl ester	$C_{18}H_{36}O_2$	284.50	0.40	Fatty acid	95.4
**34.2002**	Phytol	$C_{20}H_{40}O$	296.50	15.33	Terpenoid	96.7
**34.9009**	Phytyl acetate	$C_{22}H_{42}O_2$	338.60	6.01	Terpenoid	87.4
**35.0346**	Linoleic acid ethyl ester	$C_{20}H_{36}O_2$	308.50	0.12	Fatty acid	83.2
**35.1523**	9,12,15-Octadecatrienoic acid, ethyl ester, (Z,Z,Z)-	$C_{20}H_{34}O_2$	306.50	0.15	Fatty acid	84.7
**39.9375**	Androsta-1,4-dien-3-one, 6,17-dihydroxy-,(6.beta.,17.alpha.)-	$C_{19}H_{26}O_3$	302.40	3.63	Steroid	80.9
**45.3255**	Squalene	$C_{30}H_{50}$	410.70	3.29	Hydrocarbon	94.4
**46.3311**	Nonacosane	$C_{29}H_{60}$	408.80	2.41	Hydrocarbon	95.8
**48.2459**	D-gamma-Tocopherol	$C_{28}H_{48}O_2$	416.70	0.29	Tocopherol	85.3
**48.9145**	Tetratriacontane	$C_{34}H_{70}$	478.90	4.58	Hydrocarbon	93.8
**49.2729**	.alpha.-Tocopherol-.beta.-D-mannoside	$C_{35}H_{60}O_7$	592.80	2.08	Tocopherol	96.0
**50.7117**	Stigmasterol	$C_{29}H_{48}O$	412.70	1.30	Steroid	86.3
**51.3589**	Chondrillasterol	$C_{29}H_{48}O$	412.70	5.65	Steroid	91.0
**52.2467**	Alpha-amyrin	$C_{30}H_{50}O$	426.70	0.68	Terpenoid	87.1
**54.2632**	Phytyl palmitate	$C_{36}H_{70}O_2$	534.94	5.49	Fatty acid	91.6
**56.2101**	Phytyl linoleate	$C_{38}H_{70}O_2$	558.96	1.40	Fatty acid	83.1

RT: retention time

Supplementary Table S3. Phytochemical constituents identified in DEGE using GC-MS

RT (min)	Identified compound	Formula	Molecular mass (g/mol)	Area (%)	Compound nature	Match factor
**18.2718**	Copaene	$C_{15}H_{24}$	204.35	0.04	Hydrocarbon	86.1
**19.3789**	Caryophyllene	$C_{15}H_{24}$	204.35	0.08	Terpene	92.7
**21.8500**	2,4-Di-tert-butylphenol	$C_{14}H_{22}O$	206.32	0.23	Phenol	95.5
**22.0319**	Dihydroactinidiolide	$C_{11}H_{16}O_2$	180.24	0.26	Terpene	93.8
**27.3913**	Loliolide	$C_{11}H_{16}O_3$	196.24	0.87	Benzofuranone	96.5
**28.8889**	Phytene-2	$C_{20}H_{40}$	280.50	0.59	Hydrocarbon	88.9
**29.0013**	Neophytadiene	$C_{20}H_{38}$	278.50	11.81	Terpene	97.3
**29.4826**	Phytol, acetate	$C_{22}H_{42}O_2$	338.60	5.67	Terpenoid	95.5
**32.0126**	Hexadecanoic acid, ethyl ester	$C_{18}H_{36}O_2$	284.50	0.25	Fatty acid	95.1
**34.1963**	Phytol	$C_{20}H_{40}O$	296.50	7.66	Terpenoid	96.9
**34.8902**	Phytyl acetate	$C_{22}H_{42}O_2$	338.60	0.12	Terpenoid	84.5
**35.0293**	Ethyl linoleate	$C_{20}H_{36}O_2$	308.50	0.06	Fatty acid	82.5
**35.1416**	Ethyl linolenate	$C_{20}H_{34}O_2$	306.50	0.11	Fatty acid	86.5
**39.9073**	Androsta-1,4-dien-3-one, 6,17-dihydroxy-,(6.beta.,17.alpha.)-	$C_{19}H_{26}O_3$	302.40	1.31	Steroid	81.4
**45.3202**	Squalene	$C_{30}H_{50}$	410.70	6.72	Hydrocarbon	95.7
**46.3204**	Nonacosane	$C_{29}H_{60}$	408.80	3.95	Hydrocarbon	97.0
**46.7322**	1,6,10,14,18,22-Tetracosahexaen-3-ol,2,6,10,15,19,23-hexamethyl-, (all-E)-	$C_{30}H_{50}O$	426.72	0.28	Terpenoid	82.4
**48.2299**	D-gamma-Tocopherol	$C_{28}H_{48}O_2$	416.70	1.06	Tocopherol	95.7
**48.9145**	Tetratriacontane	$C_{34}H_{70}$	478.90	7.44	Hydrocarbon	95.2
**49.2622**	Alpha-tocopherol	$C_{29}H_{50}O$	430.70	4.19	Tocopherol	97.7
**50.7010**	Stigmasta-5,22-dien-3-ol, (3.beta.,22E)-	$C_{29}H_{48}O$	412.71	1.02	Sterol	92.5
**50.9524**	Chondrillasterol	$C_{29}H_{48}O$	412.71	7.53	Sterol	91.1
**52.2307**	Alpha-amyrin	$C_{30}H_{50}O$	426.70	0.80	Terpenoid	92.8
**54.2579**	Phytyl palmitate	$C_{36}H_{70}O_2$	534.94	12.69	Fatty acid	93.5
**56.1941**	Phytyl linoleate	$C_{38}H_{70}O_2$	558.92	1.88	Fatty acid	87.2
**56.3974**	Phytyl stearate	$C_{38}H_{74}O_2$	563.00	2.34	Fatty acid	81.6

RT: retention time

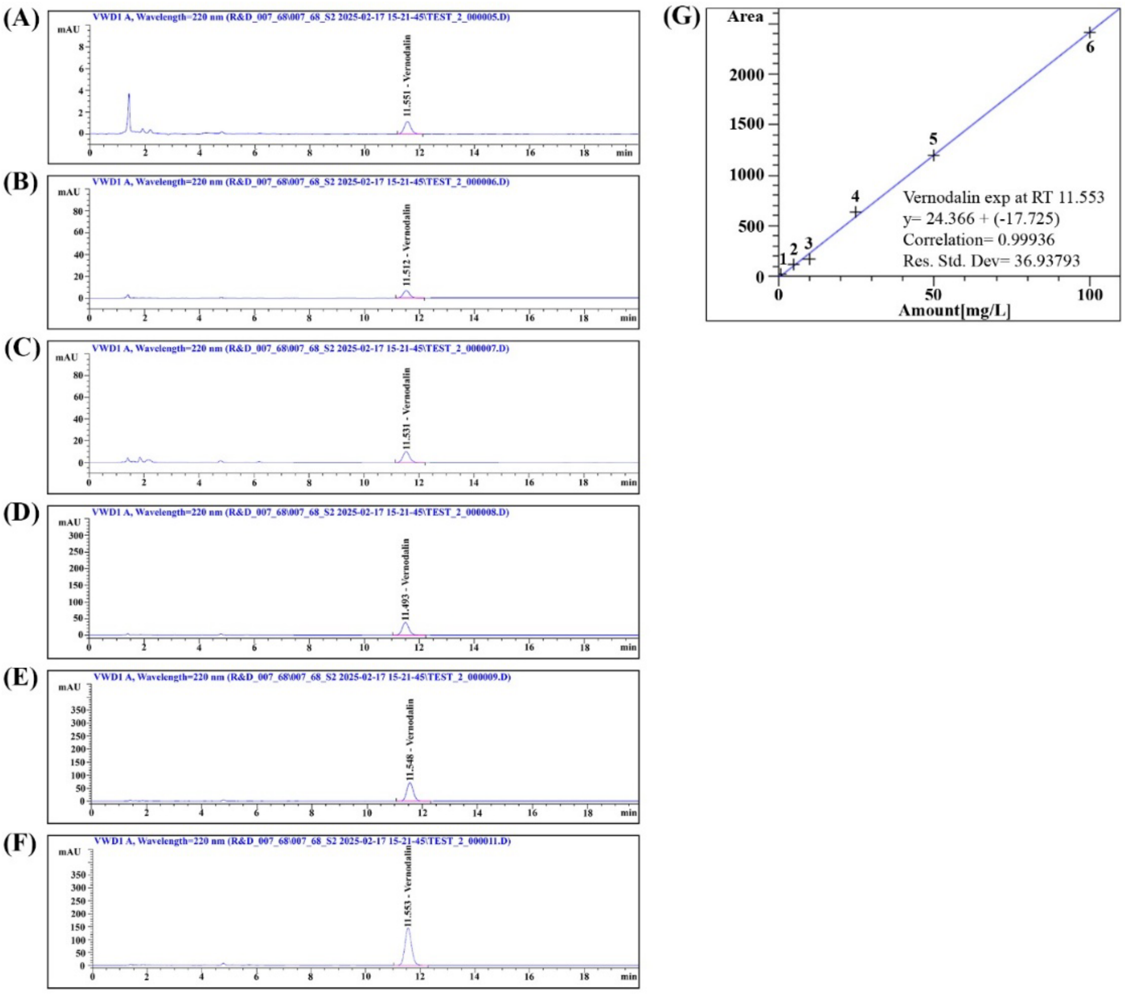

Supplementary Figure S5. HPLC chromatogram of VD standard **(A)** 1 mg/l, **(B)** 5 mg/l, **(C)** 10 mg/l, **(D)** 25 mg/l, **(E)** 50 mg/l, **(F)** 100 mg/l, **(G)** standard calibration curve

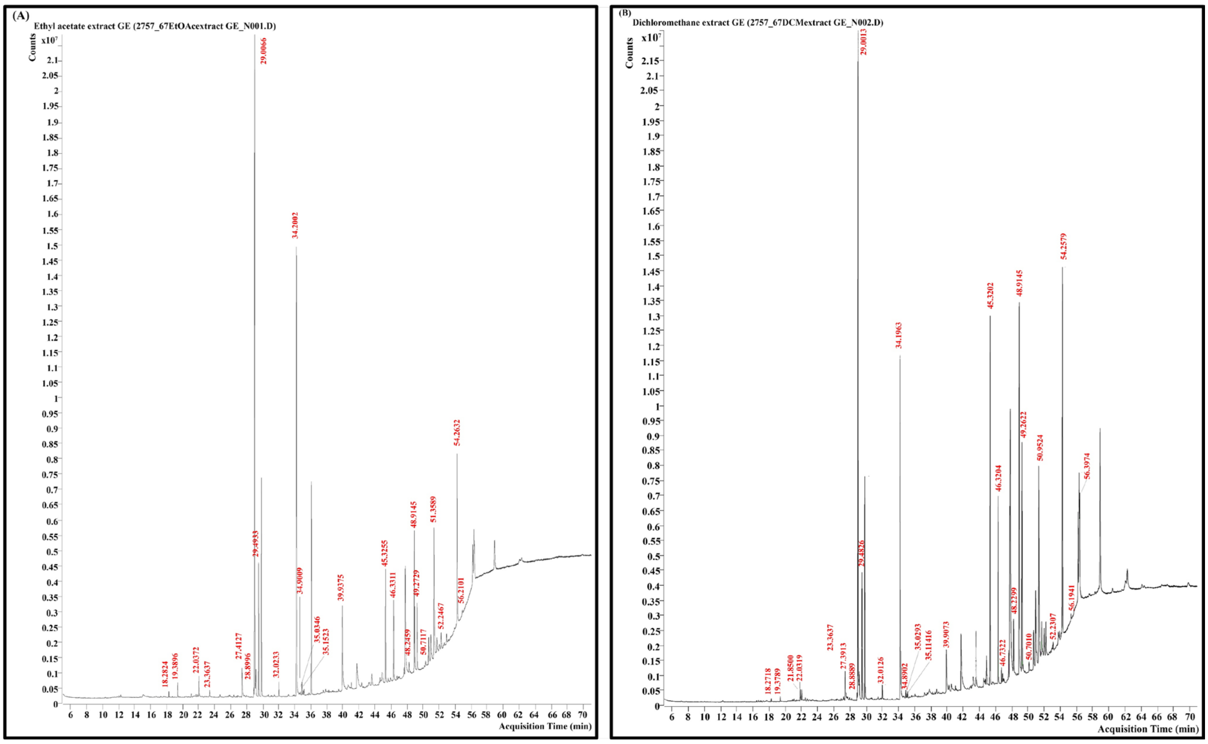

Supplementary Figure S6. GC-MS chromatograms and relative component peak areas of **(A)** EAGE and **(B)** DEGE. The chromatographic profiles illustrate the qualitative and quantitative differences in phytochemical composition between the two extracts. Individual components were identified based on mass spectral matching with the NIST library database, and peak areas are expressed as relative percentages of the total ion chromatogram.

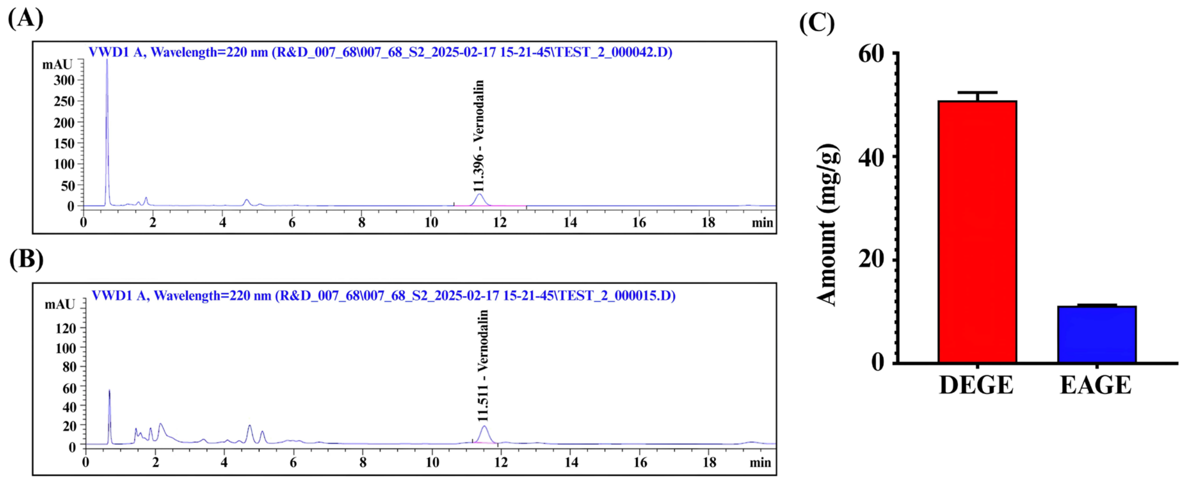

Supplementary Figure S7. HPLC chromatograms of **(A)** ethyl acetate extract (EAGE) and **(B)** dichloromethane extract (DEGE) from *G. extensum* leaves. **(C)** Quantitative analysis showing the concentration of VD in EAGE and DEGE.
